# Magnitude and determinants of obstetric case fatality rate among women with the direct causes of maternal deaths in Ethiopia: a national cross sectional study

**DOI:** 10.1186/s12884-020-2830-5

**Published:** 2020-02-27

**Authors:** Ayele Geleto, Catherine Chojenta, Tefera Taddele, Deborah Loxton

**Affiliations:** 1 School of Public Health, College of Health and Medical Sciences, Haramaya University, Harar, Ethiopia; 2 Research Centre for Generational Health and Ageing, School of Medicine and Public Health, Faculty of Health and Medicine, the University of Newcastle, Newcastle, Australia; 3 Health System and Reproductive Health Directorate, the Ethiopian Public Health Institute, Addis Ababa, Ethiopia

**Keywords:** Major direct causes, Maternal deaths, Case fatality rate, Ethiopia, Hospitals

## Abstract

**Background:**

In sub-Saharan Africa, maternal death due to direct obstetric complications remains an important health threat for women. A high direct obstetric case fatality rate indicates a poor quality of obstetric care. Therefore, this study was aimed at assessing the magnitude and determinants of the direct obstetric case fatality rate among women admitted to hospitals with direct maternal complications.

**Methods:**

In 2015, the Ethiopian Public Health Institute conducted a national survey about emergency obstetric and newborn care in which data about maternal and neonatal health indicators were collected. Maternal health data from these large national dataset were analysed to address the objective of this study. Descriptive statistics were used to present hospital specific characteristics and the magnitude of direct obstetric case fatality rate. Logistic regression analysis was performed to examine determinants of the magnitude of direct obstetric case fatality rate and the degree of association was measured using an adjusted odds ratio with 95% confidence interval at *p* < 0.05.

**Results:**

Overall, 335,054 deliveries were conducted at hospitals and 68,002 (20.3%) of these women experienced direct obstetric complications. Prolonged labour (23.4%) and hypertensive disorders (11.6%) were the two leading causes of obstetric complications. Among women who experienced direct obstetric complications, 435 died, resulting in the crude direct obstetric case fatality rate of 0.64% (95% CI: 0.58–0.70%). Hypertensive disorders (27.8%) and maternal haemorrhage (23.9%) were the two leading causes of maternal deaths. The direct obstetric case fatality rate varied considerably with the complications that occurred; highest in postpartum haemorrhage (2.88%) followed by ruptured uterus (2.71%). Considerable regional variations observed in the direct obstetric case fatality rate; ranged from 0.27% (95% CI: 0.20–0.37%) at Addis Ababa city to 3.82% (95% CI: 1.42–8.13%) at the Gambella region. Type of hospitals, managing authority and payment required for the service were significantly associated with the magnitude of direct obstetric case fatality rate.

**Conclusions:**

The high direct obstetric case fatality rate is an indication for poor quality of obstetric care. Considerable regional differences occurred with regard to the direct obstetric case fatality rate. Interventions should focus on quality improvement initiatives and equitable resource distribution to tackle the regional disparities.

## Background

Obstetric complications are a global health threat for women affecting about a sixth of all pregnant mothers [1]. An obstetric complication is defined as an acute condition arising from either a direct or indirect cause of maternal death. Direct obstetric complications include maternal complications such as maternal haemorrhage, obstructed labour, sepsis, complications of abortion, pre-eclampsia or eclampsia, ectopic pregnancy, postpartum sepsis, and ruptured uterus [2]. Indirect obstetric complications are the exacerbation of pre-existing maternal health conditions or illnesses including anaemia, malaria and other health conditions, that a woman acquires during pregnancy and childbirth and are not directly caused by the pregnancy [3]. Unless appropriately managed, several obstetric complications that arise during pregnancy and childbirth can end in maternal deaths [3]. Therefore, reduction of maternal mortality has become the top global health priority that can be achieved through the provision of quality obstetric care [4].

Reports from various countries show that maternal deaths remain the most important public health problem in sub-Saharan Africa [5]. A recent report jointly released by the WHO, UNICEF, UNFPA, World Bank Group, and the United Nations Population Division revealed that the global MMR had declined by 38.4% from 2000 to 2017 with an average annual reduction rate of 2.9%. According to this report, although sub-Saharan Africa achieved a substantial reduction, still MMR remains at higher magnitude than other regions. In Ethiopia, MMR was 401 per 100,000 live births in 2017 [6]. With its current MMR, Ethiopia is one of the countries with the highest MMR and almost all of these deaths are accounted to the direct obstetric complications [7]. Almost all (99%) of the global maternal mortality has occurred in low-income countries and 66% of these deaths are accounted for by countries in sub-Saharan Africa [8].

Direct obstetric case fatality rate (DOCFR) is defined as the proportion of women who are admitted to a hospital or an emergency obstetric care facility with direct obstetric complications, or who develop such conditions after admission, and die before discharge [9]. The WHO recommends the DOCFR as a useful crude indicator of the quality of obstetric care provided at a particular health facility. The DOCFR is estimated in such a way that the number of women who experienced direct obstetric complications is used as the denominator and the numerator being the number of women who died due to direct obstetric complications. Accordingly, obstetric services are said to be of good quality if the DOCFR is less than 1% [10]. However, several study findings in most sub-Saharan African countries show a higher DOCFR than the recommended threshold. For example, researchers in Nigeria showed that, DOCFR was 3.2% [11] while findings of a study in Malawi revealed that DOCFR was 2.0% [12]. In Ethiopia, the magnitude of DOCFR for women with major direct obstetric complications was reported to be 2% [13].

Ethiopia is one of the countries in the Horn of Africa with a population of different cultures, ethnicities and religions [14]. According to the United Nations Worldometers (2019), the total population of Ethiopia was 109,302,118 as at March 2019. Ethiopia is the twelfth most populous country in the world, and the second in Africa, next to the Nigeria [15]. Currently, the total fertility rate is 4.6, the contraceptive prevalence rate for currently married women is 36%, and the unmet need for family planning is 22% [7]. Only 74% pregnant women visited a health facility for the first antenatal care while only 43% of pregnant mothers visited a facility four times. Only 34% of women were reported as meeting the Safe Motherhood Programmes’ recommendation of receiving a postnatal care check within two days of delivery [16].

Evidence about DOCFR is assumed the better indicators for designing, monitoring and evaluating the quality of maternal health interventions. In Ethiopia, despite the high maternal mortality rate, limited literature exists about the DOCFR. To the authors’ knowledge, determinants of the DOCFR has not been studied in Ethiopia. Therefore, the aim of this study was to assess the magnitude of the DOCFR and its determinants among women admitted to hospitals in Ethiopia.

## Methods

### Study area and period

This study utilized a large public dataset available at the Ethiopian Public Health Institute (EPHI). The EPHI is a large-scale research institute, which undertakes various basic and operational researches on the priority health agenda to generate and disseminate scientific knowledge that help in improving the health of the population. The EPHI conducted a national cross-sectional survey titled ‘assessment of emergency obstetric and newborn care in Ethiopia’ in 2016. With this project, different data about maternal and neonatal health indicators were collected from health facilities’ logbooks.

### Study participants

In the 2016 national survey, the EPHI included all public and private health facilities found in the country, which were deemed functional and offered delivery services. There were 3804 health facilities that were included in the survey to collect data about maternal and neonatal health indicators. However, the current study analysed the data of all governmental/public and private hospitals, of which there were 293 at the time of data collection. The data that were collected from 30 (10.2%) specialized hospitals, 103 (35.2%) general hospitals and 160 (54.6%) primary hospitals were included.

### Inclusion and exclusion criteria

The data about number and type of obstetric complications, maternal deaths and all deliveries conducted throughout 2015 among all hospitals found in Ethiopia were included in the analysis. The data of all public/government and private hospitals were included with the exclusion of maternal data collected from the lower level facilities including clinics and health centres. In Ethiopia, women who have experienced obstetric complications are often referred to hospitals where they can receive specialized treatment. Therefore lower level health facilities were not included in the analysis since information of the majority of women who sustained obstetric complications can be obtained from hospitals.

### Nature of the data

The EPHI collected data about the performance of the Emergency Obstetric and Newborn Care (EmONC) signal functions at a particular hospital using a standardized questionnaire. The EPHI collected the data from hospitals’ registers and records within the reference period of January–December 2015 inclusive. The available dataset included the number and mode of deliveries, number of women who experienced different types of direct obstetric complications and associated maternal deaths that occurred in each hospital. The dataset also had different facility-specific characteristics including type of hospital, managing authority, location of the hospitals and fulfilment of basic facility infrastructure.

The primary outcome variable is the DOCFR, the proportion of women who are admitted to a hospital or an emergency obstetric care facility with major direct obstetric complications, or who develop such complications after admission, and die before discharge [9]. Then hospitals’ specific characteristics such as type, managing authority, and location of the hospitals and fulfilment of basic facility infrastructure were assessed for whether they predict the magnitude of the DOCFR in hospitals or not.

### Operational definitions

- Major direct obstetric complications: In this study, the following were considered as major direct obstetric complications: Antepartum Haemorrhage (APH), Postpartum Haemorrhage (PPH), retained placenta, prolonged/obstructed labour, postpartum sepsis (infections that a women acquire during labour, childbirth and immediate postpartum period), ruptured uterus, severe eclampsia/preeclampsia, complications of abortion and ectopic pregnancy [9].
- Other direct obstetric complications: These complications include premature rupture of membrane, post-term labour, cord prolapse, breech presentation, and other possible problems that were not considered as a major direct cause of maternal deaths [17].
- Direct Obstetric Case Fatality Rate (DOCFR): the proportion of women who are admitted to a hospital or an EmOC facility with the major direct obstetric complications or who develop such complications after admission and die before discharge [9].
- DOCFR ≥ 1: is a crude indicator for poor quality of obstetric services provided at a particular health facility [10].

### Data analysis

Initially, descriptive statistics including means, percentages, frequency tables and ratios were used to describe facility specific characteristics and the magnitude of the DOCFR. Then logistic regression analysis was conducted to examine the association between the DOCFR and hospital-specific characteristics. Maternal deaths due to direct obstetric complications that were reported in all hospitals during 2015 were included to determine the magnitude of the DOCFR. For the analysis purpose, the magnitude of the DOCFR was calculated for each hospital according to the WHO definition, the proportion of women admitted to a hospital or an EmOC facility with direct obstetric complications, or who developed such complications after admission, and died before discharge [9]. Using the number of women who experienced direct obstetric complications as the denominator, we calculated the DOCFR, the numerator being the number of maternal deaths attributed to direct obstetric causes. Hospitals were dichotomized based on the WHO recommendation of DOCFR: hospitals with DOCFR ≥1 (hospitals with unfavourable outcomes) and hospitals with DOCFR < 1 (hospitals with favourable outcomes). First, we conducted univariate logistic regression analysis to assess the presence of association between the magnitude of the DOCFR and hospital-specific characteristics. Facility-level variables that were significantly associated with the magnitude of the DOCFR in the univariate analysis were successively included in a multivariate model, using a backwards-stepwise elimination to select variables. For all models fitted, we used generalised linear model for the multilevel analysis by using Stata version 15 statistical software. The degree of association between variables was measured using an adjusted odds ratio with 95% confidence interval and association was declared significant at *p* value < 0.05.

### Ethical clearance

The Ethiopian national survey titled ‘Assessment of emergency obstetric and newborn care in Ethiopia’ was granted ethical approval from the Scientific and Ethical Review Office of the EPHI (approval number: EPHI-6-13-728) on 6-Jun-2016. Ethical approval for this analysis was obtained from the Human Research Ethics Committee (HREC) of The University of Newcastle, Australia (approval number: H-2018-0245) on 15-Aug-2018. The protocol developed to conduct this analysis had received ethical approval from the Scientific and Ethical Review Office of the EPHI before the data were accessed (Protocol number: EPHI-IRB-048-2018) on 25-July-2018.

## Results

### Hospitals characteristics

The current study presented the magnitude of DOCFR and associated factors in 293 hospitals found in nine regional states and two city administrations of Ethiopia. Nearly a fourth (N = l73; 24.9%) of the hospitals were from Oromia region and 30 (10.2%) of the hospitals were referral specialized hospitals. Two hundred and thirty-five (80.2%) hospitals were public hospitals while the remaining were private facilities. Geographically, 272 (92.8%) hospitals were located in urban centres. The majority of the hospitals, (*N* = 207; 70.6%) had initiated the maternal death review and reporting system (MDRS); a health reform used to continuously notify, review, analyse and respond to maternal deaths (Table 1).

**Table 1:** Characteristics of hospitals included in the study, Ethiopia, 2015

Variables	Number of hospitals	Percent
Region		
Addis Ababa	33	11.3
Afar	6	2.0
Amhara	56	19.1
Benishangul Gumz	3	1.0
Dire Dawa	6	2.0
Gambella	1	.3
Harari	6	2.0
Oromia	73	24.9
SNNPR	60	20.5
Somali	10	3.4
Tigray	39	13.3
**Type of hospital**		
Referral/specialized	30	10.2
General hospital	103	35.2
Primary hospital	160	54.6
**Managing authority**		
Public/Government	235	80.2
Private	58	19.8
**Location**		
Urban	272	92.8
Rural	21	7.2
**Availability of electricity**		
No	12	4.1
Yes	281	95.9
**Availability of water**		
No	10	3.4
Yes	281	95.9
**Availability of functional toilet**		
No	8	2.7
Yes	285	97.3
**Formal payment required for the service**^**a**^		
No	89	30.4
Yes	204	69.6
**Has the hospital implemented MDRS**		
No	86	29.4
Yes	207	70.6

*MDRS* Maternal Death Review and Reporting System

^a^: In Ethiopia, all maternal health services are given free of charge in public health facilities. However, some hospitals might request women to purchase some medical supplies and drugs

### Direct obstetric complications and associated maternal deaths

**Fig. 1:**
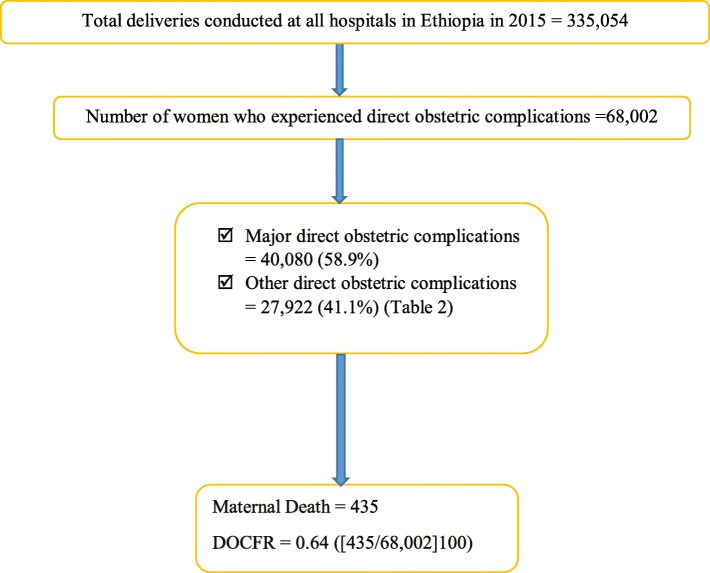
The number of childbirth, mode of delivery and number of women who experienced obstetric complications at hospitals in Ethiopia, 2015. SVD: Spontaneous Vaginal Delivery; AVD: Assisted Vaginal Delivery; CS: Caesarean Section; RU: Ruptured Uterus Major direct obstetric complications: constitutes antepartum haemorrhage, postpartum haemorrhage, retained placenta, obstructed labour, postpartum sepsis, ruptured uterus, hypertensive disorders, complications of abortion and ectopic pregnancy. Other direct obstetric complications: These include premature rupture of membrane, postterm labour, cord prolapse, breech presentation, and other possible problems that were not considered as a major direct obstetric complication

Overall, 335,054 deliveries were conducted in all hospitals in Ethiopia and 68,002 women experienced direct obstetric complications, giving the crude incidence rate of 203 per 1000 deliveries (68,002/335054). Major direct obstetric complications accounted for 40,080 (58.9%) of the cases while 27,922 (41.1%) of women experienced other direct obstetric complications (Fig. 1). Prolonged labour was the leading cause of major direct obstetric morbidity, accounting for 23.4% of the total cases. Hypertensive disorders was the second most frequent cause of major direct maternal morbidity as 11.6% of mothers experienced this condition (Table 2).

**Table 2:** Magnitude of direct obstetric complications and associated maternal death among hospitals in Ethiopia, 2015

Direct obstetric complications	Number of cases (%)	Direct obstetric complication rate^a^	Number of death (%)	DOCFR in % (95%CI)
Antepartum Haemorrhage	4462 (6.5)	13.3 (12.9–13.7)	23 (5.3)	0.51 (0.32–0.77)
Postpartum Haemorrhage	2811 (4.1)	8.4 (8.1–8.7)	81 (18.6)	2.88 (2.29–3.57)*
Retained placenta	2201 (3.2)	6.5 (6.3–6.8)	4 (0.9)	0.18 (0.04–0.46)
Prolonged labour	15,875 (23.4)	47.4 (46.7–48.2)	25 (5.7)	0.16 (0.10–0.23)
Rupture of uterus	1516 (2.2)	4.5 (4.3–4.7)	41 (9.5)	2.71 (1.95–3.65)*
Postpartum sepsis	1460 (2.2)	4.3 (4.1–4.6)	17 (3.9)	1.16 (0.67–1.85)
Hypertensive disorder	7912 (11.6)	23.6 (23.1–24.2)	121 (27.8)	1.53 (1.27–1.82)*
Abortion complications	2042 (3.0)	6.1 (5.8–6.4)	6 (1.5)	0.29 (0.11–0.64)
Ectopic pregnancy	1801 (2.7)	5.3 (5.1–5.6)	0 (0.0)	–
Other direct causes	27,922 (41.1)	83.5 (82.5–84.4)	117 (26.8)	0.42 (0.34–0.50)
Total	68,002 (100)	203.3 (201.9–204.7	435 (100)	0.64 (0.58–0.70)

*DOCFR* Direct Obstetric Case Fatality Rate

*statistically significantly greater DOCFR than 1% (the recommended threshold by the World Health Organization); *p* < 0.05

-: there were no maternal deaths due to ectopic pregnancy hence DOCFR was not calculated

^a^Direct obstetric complication rate was estimated by the complications per 1000 births

Of women who experienced direct obstetric complications, 435 died, resulting in crude national DOCFR of 0.64 (95% CI: 0.58–0.70) per 100 mothers (435/68,002). That is, a general ratio of 1 death for every 156 cases of direct obstetric complications was observed. Hypertensive disorders was the leading cause of direct maternal mortality, accounting for 27.8% of the overall maternal deaths. Obstetric haemorrhage was the second most frequent direct cause of maternal mortality, accounting for 104 (23.9%) of the overall maternal deaths. The majority of deaths due to obstetric haemorrhage (*N* = 81; 77.8%) were caused by postpartum haemorrhage. On the other hand, postpartum haemorrhage accounted for 18.6% of the overall maternal deaths. The DOCFR varied considerably with the complications that occurred. The highest DOCFR was observed in postpartum haemorrhage 2.88% (95% CI: 2.29–3.57%) followed by ruptured uterus 2.71% (95% CI: 1.95–3.65%). The DOCFR due to hypertensive disorders 1.53% (95% CI: 1.27–1.82%) and postpartum sepsis 1.16% (95% CI: 0.67–1.85%) were also in excess of the recommended level (Table 2).

### Regional variations in the direct obstetric case fatality rate

Although the crude national DOCFR was within the recommended threshold of less than 1%, considerable regional variation occurred. The DOCFR ranged from 0.27% (95% CI: 0.20–0.37%) in Addis Ababa city administration to 3.82% (95% CI: 1.42–8.13%) in the Gambella region. The DOCFR was significantly higher in the Afar 1.39 (95% CI: 0.45–3.22), the Harari 1.08 (95%CI: 0.61–1.78) and the Somali 1.69 (95% CI: 1.13–2.42) regions (Table 3).

**Table 3:** Regional distribution of direct obstetric case fatality rate among hospitals in Ethiopia, 2015

Regions in Ethiopia	Number of women with complications	Maternal deaths due to the complication (n)	DOCFR in percent with 95% CI
Addis Ababa	14,453	39	0.27 (0.20–0.37)
Afar	358	5	1.39 (0.45–3.22)
Amhara	11,263	89	0.79 (0.63–0.97)
Benishangul Gumuz	1302	4	0.30 (0.08–0.78)
Dire Dawa	1437	8	0.55 (0.24–1.09)
Gambella	157	6	3.82 (1.42–8.13)*
Harari	1384	15	1.08 (0.61–1.78)
Oromia	17,084	128	0.74 (0.62–0.89)
SNNPR	12,942	82	0.63 (0.50–0.78)
Somali	1710	29	1.69 (1.13–2.42)*
Tigray	5912	30	0.51 (0.34–0.72)
**Total**	**68,002**	**435**	**0.63 (0.58–0.70)**

*SNNPR* Southern Nations Nationalities and People Region

*DOCFR* Direct Obstetric Case Fatality Rate

*statistically significantly greater DOCFR than 1% (the recommended threshold by the World Health Organization); *p* < 0.05

### Determinants of the direct obstetric case fatality rate among hospitals

Bivariate and multivariate logistic regression analyses were conducted to analyse the hospital specific determinants of the magnitude of DOCFR. In the final multivariate analysis, whether formal payment was required for the service or not, types and managing authority of the hospitals were found to be significantly associated with the magnitude of DOCFR after possible confounders were controlled for. The odds of observing DOCFR≥1 was reduced by 58% in general hospitals as compared to primary hospitals [AOR = 0.42; 95%CI (0.21, 0.85)]. The magnitude of DOCFR was also lower in specialized hospitals compared to primary hospitals although the difference was not statistically significant. Regarding managing authorities, the odds of DOCFR≥1 was about two times higher in public/governmental hospitals than private counterparts [AOR = 2.46; 95% CI (1.28, 4.32)]. Payment was also identified as a factor in that the odds of DOCFR≥1 was 64% higher in hospitals where formal payment was not required for the service than in hospitals where mothers need to cover service fee [AOR = 1.64; 95%CI (1.04, 3.19)]. This might be explained by differences in quality of care between public and private hospitals. In private hospitals where clients need to cover costs associated with health service, better quality of obstetric care were provided than it was in public hospitals (Table 4).

**Table 4:** Unadjusted and adjusted logistic regression model of factors associated with maternal deaths among hospital in Ethiopia, 2015

Variables	Responses	DOCFR		Unadjusted Odds ratio	Adjusted Odds ratio	*P* value
		≥1	< 1			
Type of hospitals	Referral hospital	5	25	0.83 (0.29, 2.41)	0.97 (0.33, 2.93)	0.964
	General hospital	25	78	0.27 (0.27, 0.98)	**0.42 (0.21, 0.85)**	**0.016**
	Primary hospital	23	137	Ref	Ref	
Managing authority	Public/Government	49	186	3.55 (1.27, 9.86)	**2.16 (1.28, 4.32)**	**0.032**
	Private	4	54	Ref	Ref	
Location	Rural	5	16	1.45 (0.52, 4.03)	0.87 (0.21, 3.53)	0.851
	Urban	48	224	Ref	Ref	
Have electric supply	No	1	11	0.40 (0.05, 3.17)	1.11 (0.07, 15.77)	0.939
	Yes	52	229	Ref	Ref	
Have Water supply	No	3	7	1.98 (0.49, 7.31)	3.11 (0.50, 19.35)	0.223
	Yes	50	231	Ref	Ref	
Availability of functional toilet	No	3	5	2.82 (0.65, 11.08)	2.22 (0.45, 10.89)	0.323
	Yes	50	235	Ref	Ref	
Payment required for the service	No	23	30	2.02 (1.09, 3.72)	**1.64 (1.04, 3.19)**	**0.023**
	Yes	66	174	Ref	Ref	
System in place to waive service fee	No	25	133	0.71 (0.38, 1.28)	0.79 (0.41, 1.52)	0.490
	Yes	28	105	Ref	Ref	
The hospital implement MDSR	No	6	80	0.25 (0.10, 0.62)	0.24 (0.09, 1.18)	0.06
	Yes	47	160	Ref	Ref	

*DOCFR* Direct Obstetric Case Fatality Rate, *MDRS* Maternal Death Review and Reporting System

Hospitals were dichotomized based on the WHO recommendation of DOCFR: hospitals with DOCFR ≥1 (unfavourable DOCFR or hospitals with the outcome of interest) and hospitals with DOCFR < 1 (favourable DOCFR or hospitals without the outcome of interest)

## Discussion

This study evaluated the DOCFR and determinant factors among women who were admitted to Ethiopian hospitals with direct maternal morbidity in 2015. Overall, the DOCFR among women who were admitted to hospitals was found to be 0.64% (435/68,002). Prolonged labour was the leading cause of direct obstetric morbidity followed by hypertensive disorders. Hypertensive disorders of pregnancy, maternal haemorrhage, ruptured uterus and prolonged labour were the predominant direct causes of maternal deaths. The DOCFR was significantly higher among mothers who were admitted with postpartum haemorrhage and ruptured uterus. The DOCFR was also unacceptably high among mothers who experienced hypertensive disorders and sepsis. Significant regional variation was observed in the DOCFR, the highest being occurred in the Gambella region while the lowest was observed in Addis Ababa city administration, the capital city of Ethiopia.

The current study showed that in 2015, a large proportion (20.3%) of women who gave birth in hospitals experienced the direct obstetric complications (68,002/335,054). This finding is unsurprising as an estimated 15% of all pregnancies ended in obstetric complications [1]. Consistent with findings of an Eritrean study [18], our finding revealed that hypertensive disorders of pregnancy were the leading cause of maternal mortality. Similarly, our study revealed that postpartum haemorrhage, prolonged labour and postpartum sepsis were the predominant causes of maternal mortality. Our finding is supported by Brazil’s findings [19], findings in Kenya [20] and several small scale studies conducted in Ethiopia [21–23].

This study demonstrated that the crude national DOCFR was within the WHO recommended level of less than 1% [9]. A significant reduction in the DOCFR was observed as compared to findings of a previous study where the national obstetric case fatality rate was 2% in 2008 [13]. This reduction might be explained by improved access to maternal health service, as the government of Ethiopia strengthened the health system while striving to achieve the Millennium Development Goals (MDGs) in reducing maternal mortality. The current finding is consistent with a study conducted in Indonesia, both in the magnitude and trends of the DOCFR [24].

Nevertheless, the current study demonstrated a considerable variation in the DOCFR among different direct causes of maternal deaths. Postpartum haemorrhage carried the highest DOCFR followed by ruptured uterus. The DOCFR for hypertensive disorders and postpartum sepsis also exceeded the recommended level. Our finding is consistent with the results of a systematic review conducted in sub-Saharan Africa where the case fatality rate of maternal haemorrhage, ruptured uterus, sepsis, and obstructed labour were higher than the recommended level [25]. Unlike our findings, a study conducted in six west African countries revealed higher DOCFR for sepsis (33.3%), ruptured uterus (30.4%) and hypertensive disorders of pregnancy (18.4%) [17]. This variation might be attributed to the differences in sample size and study settings. While study of the West African countries assessed the DOCFR in sample of women, our study included data of all women that our findings were highly representative.

Despite lower DOCFR at the national level, a significant regional variation was observed. The highest DOCFR was observed in the Gambella region where case fatality rate was 3.82% (95% CI: 1.42–8.13%). A considerably high DOCFR was found in the Afar, Harari and Somali regions, the lowest being in Addis Ababa city administration; the capital city of Ethiopia. Our finding is supported with the study conducted in six west African countries where the case fatality rate significantly varied among the regions involved in the study [17]. The observed variation might be occurred due to the limited access to maternal health service in the Gambella, Afar and Somali regions as these regions are found in most pastoral area of the country. Higher DOCFR than the recommended level was noted in the Harari region one of the urban settings with better access to health service. This might be attributed to poor quality of obstetric care among hospitals found in the region. The observed regional disparities in the DOCFR might also be attributed to the existing regional differences in the use of antenatal care services [26] and the low quality of antenatal care in Ethiopia [27]. The existing spatial differences of contraceptive use among Ethiopian regions could also be a cause for the observed disparities in the DOCFR among regions [28].

In the multivariate logistic regression analyses, several hospital-specific factors were significantly associated with the magnitude of the DOCFR. In the final model, the likelihood of observing DOCFR≥1 was significantly reduced among general hospitals than it was in primary hospitals. The odds of having one or higher DOCFR was also decreased among specialized hospitals though the reduction was not statistically significant. The probability of maternal death from obstetric complications is usually affected with the type and quality of management provided at the facility. According to the three tier healthcare delivery system of Ethiopia, since specialized and general hospitals are more equipped with medical supplies and qualified healthcare providers than primary hospitals [29], this finding might not be surprising.

Although the difference is not statistically significant, the odds of having DOCFR≥1 was reduced by 75% among hospitals that did not implement the MDSR initiatives. The MDSR is a health reform used to continuously notify, review, analyse and respond to maternal deaths in order to take action to prevent similar deaths in the future [30]. Although, MDSR was accepted at subnational level in many countries, it is not adequately institutionalised [31]. Several barriers including role confusion, high staff turnover and lack of necessary facilities impeded the implementation of the MDSR reform [32]. Furthermore, the observed association might reflect the situation of private hospitals where quality of the service is better but the reform was not yet well institutionalized.

However, the odds of having DOCFR≥1 among public hospitals was two times higher as compared to private hospitals counterparts. This might be explained by the better health service utilization rate at private facilities than public counterparts [33]. This difference might also be observed due to better quality of the service as more standardized maternal health service is provided in private sector [34]. The observed difference in the magnitude of death might also be attributed to that most of the private hospitals (faith based and missionary) are mainly located in rural areas where women can accessed to treatment before sequel to severe complications [35].

The current study showed higher odds of having DOCFR≥1 among hospitals where formal payment for service was not required. Studies indicated that officialising the user charges creates financial barriers and reduces utilisation of maternal health service [36]. Access to the maternal health services is also limited with an informal payments for purchasing essential medicines [37]. On the other hand, introduction of user fee exemption reforms increased maternal health services utilization [38]. Improved access to the health service of good quality significantly decreases the maternal mortality rate [39]. Higher risk of DOCFR in hospitals where service are rendered for free might be attributed to the higher maternal mortality in public hospitals where maternity services are rendered free of charge as opposed to private hospitals.

### Strength and limitation of the study

This study has several strengths. In this study, we used a national representative data collected from all hospitals found in Ethiopia. Therefore, the findings of this study are highly generalizable and can apply to all regions of Ethiopia. These findings might also be useful for other low-income countries with similar demographic and economic characteristics. From the commencement of the survey, experts from national and international partners were involved in the data collection and management processes hence the analysed data were of high quality. In the meantime, this study was suffered from the usual limitation of a cross-sectional study in that the causal relationship could not be concluded. This study did not investigate the maternal morbidity and mortality happened at the lower level of care including health centers and clinics. Although the majority of women who experienced obstetric complications are often referred to hospitals, few women might sometimes seek care from lower level facilities. Therefore, the findings of this study can only be generalized to hospitals in the country. The maternal deaths reported in this study represent only the numbers, which were registered on the hospital’s logbook. Therefore, the number of deaths might be affected by maternal deaths that happened at home, which accounts for the majority of maternal deaths in low-income countries. Similarly, we faced difficulties in comparing our findings with similar reports since there were limited similar studies available in Ethiopia.

## Conclusions

This study revealed that the overall national DOCFR among hospitals was within the recommended threshold of less than 1%, although considerable variation was observed among the different direct causes. Certain obstetric complications such as postpartum haemorrhage, ruptured uterus, sepsis, and hypertensive disorders carried a higher risk of death for women. Considerable regional differences occurred with regard to the DOCFR. Variation in the DOCFR among different regions of Ethiopia suggests that quality of obstetric care provided at hospitals of some regions might be unsatisfactory. Therefore, the government of Ethiopia needs to focus on quality improvement initiatives by taking regional variations in to consideration. Equitable resource distribution needs to be in place to tackle the regional disparities that exist. Further studies with a strong design should be conducted to investigate the relationship between different obstetric morbidity, the level of care and the individual risk factors that determine maternal deaths of the direct obstetric complications. Future studies should also include home-based deliveries and maternal morbidity and mortality that occurred at lower level of care to ensure representativeness.

## Data Availability

The data that support the findings of this study are available from EPHI but restrictions apply to the availability of these data, which were used under license for the current study, and so are not publicly available. Data are however available from the authors upon reasonable request and with permission of the EPHI.

## Abbreviations

APH: Antepartum Haemorrhage
AVD: Assisted Vaginal Delivery
CS: Caesarean Section
DOCFR: Direct Obstetric Case Fatality Rate
EmONC: Emergency Obstetric and Newborn Care
EPHI: Ethiopian Public Health Institute
HREC: Health Research Ethics Committee
IRB: Institutional Review Board
MDRS: Maternal Deaths Review and Report
MMR: Maternal Mortality Rate
PPH: Postpartum Haemorrhage
SVD: Spontaneous Vaginal Delivery
WHO: World Health Organization
